# Определение кальцитонина в смывах из пункционной иглы в диагностике первичного или метастатического медуллярного рака щитовидной железы

**DOI:** 10.14341/probl13236

**Published:** 2023-06-30

**Authors:** З. Т. Зураева, Л. В. Никанкина, Г. С. Колесникова, Ф. М. Абдулхабирова, Д. Г. Бельцевич, Н. М. Малышева, А. А. Михеенков

**Affiliations:** Национальный медицинский исследовательский центр эндокринологии; Национальный медицинский исследовательский центр эндокринологии; Национальный медицинский исследовательский центр эндокринологии; Национальный медицинский исследовательский центр эндокринологии; Национальный медицинский исследовательский центр эндокринологии; Национальный медицинский исследовательский центр эндокринологии; Национальный медицинский исследовательский центр эндокринологии

**Keywords:** медуллярный рак щитовидной железы, кальцитонин, смыв из пункционной иглы на кальцитонин, тонкоигольная аспирационная биопсия, новообразования щитовидной железы

## Abstract

**ОБОСНОВАНИЕ:**

ОБОСНОВАНИЕ. Определение кальцитонина в смывах из пункционной иглы является перспективным методом диагностики медуллярного рака щитовидной железы (МРЩЖ).

**ЦЕЛЬ:**

ЦЕЛЬ. Оценить диагностическую значимость определения кальцитонина в смывах из пункционной иглы при сравнении с цитологическим исследованием в диагностике и локализации первичного или метастатического МРЩЖ.

**МАТЕРИАЛЫ И МЕТОДЫ:**

МАТЕРИАЛЫ И МЕТОДЫ. Проведена ретроспективная оценка данных 67 пациентов с подозрительными узловыми изменениями щитовидной железы и/или лимфатическими узлами, находившихся на лечении в ФГБУ «НМИЦ эндокринологии» в период с 2015 по 2020 гг. Первичной конечной точкой исследования была оценка диагностической точности определения кальцитонина в смывах из пункционной иглы при сравнении с изолированным цитологическим исследованием. Вторичной конечной точкой было определение оптимального диагностического уровня для использования в клинической практике.

**РЕЗУЛЬТАТЫ:**

РЕЗУЛЬТАТЫ. Результаты нашего исследования показали, что во всех случаях гистологически верифицированного МРЩЖ отмечаются повышенные уровни кальцитонина в смыве из пункционной иглы (ТАБ-КТ) как при первичных формах (чувствительность и специфичность 100%), так и при метастазах МРЩЖ в лимфатические узлы (чувствительность 88,5% и специфичность 100%). Оптимальный диагностический уровень для значений ТАБ-КТ из узлов щитовидной железы составил >122 пг/мл, из лимфатических узлов — >35,8 пг/мл.

**ЗАКЛЮЧЕНИЕ:**

ЗАКЛЮЧЕНИЕ. Определение кальцитонина в смывах из пункционной иглы является перспективным верифицирующим методом в дополнение к цитопатологическому исследованию в диагностике как первичных, так и рецидивирующих форм МРЩЖ. Для валидации диагностических значений ТАБ-КТ требуется проведение более крупных клинических исследований.

## Abstract (en)

**BACKGROUND:**

BACKGROUND: The diagnostic value of calcitonin measurement in fine-needle aspiration biopsy wash-out fluid is a promising tool in the diagnosis of medullary thyroid cancer.

**AIMS:**

AIMS: We assessed the potential usefulness and the diagnostic significance of Ct-FNAB alone in comparison with cytology in the diagnosis and localization of primary or metastatic MTC.

**MATERIALS AND METHODS:**

MATERIALS AND METHODS: For this purpose, we retrospectively examined data from 67 patients with suspicious thyroid nodules and/or lymph nodes who ultimately underwent surgical treatment at the Endocrinology Research Centre in 2015–2020. The primary endpoint of the study was to evaluate the diagnostic accuracy of Ct-FNAB when compared to cytological examination. The secondary endpoint was to determine the optimal diagnostic level for use in clinical practice.

**RESULTS:**

RESULTS: The obtained results showed that high Ct-FNAB concentrations were present in all histologically proven MTC, either in thyroid gland (sensitivity 92.5%, specificity 100%) or neck masses (sensitivity 88.5%, specificity 100%). The optimal diagnostic threshold for Ct-FNAB values from thyroid nodes was > 122 pg/ml, from lymph nodes >35.8 pg/ml.

**CONCLUSIONS:**

CONCLUSIONS: Our findings suggest that Ct-FNAB is a highly reliable diagnostic procedure to identify primary and recurrent/metastatic MTC. The actual relevance of this technique in the management of MTC needs further longitudinal studies in a larger number of patients.

## ОБОСНОВАНИЕ

Медуллярный рак щитовидной железы (МРЩЖ) составляет всего 4–6% в структуре всех злокачественных образований щитовидной железы и имеет более агрессивное течение по сравнению с дифференцированными формами карцином, составляя 8–15% причин смерти в структуре общей смертности от рака щитовидной железы [1][2]. Спорадические формы составляют примерно 75–80% всех случаев МРЩЖ, оставшиеся 20–25% встречаются в составе аутосомно-доминантных наследственных синдромов в результате герминальных мутаций в протоонкогене RET (REarranged during Transfection; синдром множественных эндокринных неоплазий (МЭН) типа 2А (90–95%) и 2В (5–10%)) [3]. Гистологическая стадия процесса и возраст пациента на момент диагностики заболевания являются наиболее важными факторами, определяющими прогноз заболевания. 10-летняя выживаемость пациентов с МРЩЖ на I, II, III и IV стадиях составляет 100, 93, 71 и 21% соответственно [3]. Центральное и латеральное метастазирование МРЩЖ наблюдается у 14 и 11% пациентов на стадии I соответственно и у 86 и 93% на стадии IV [4].

В настоящее время достигнуты значительные успехи в диагностике заболевания благодаря комплексному использованию биохимических, инструментальных и цитологических методов исследования, что в ряде случаев позволяет диагностировать патологию на самых ранних стадиях и избежать ее прогрессирования [3].

Основными методами диагностики МРЩЖ являются определение сывороточной концентрации базального и стимулированного кальцитонина, а также цитологическое исследование пункционного материала. Однако каждый из указанных методов обладает рядом ограничений, лимитирующих их диагностическую точность.

Определение кальцитонина в качестве скринингового метода у пациентов с узловыми изменениями щитовидной железы позволило улучшить раннюю диагностику заболевания и повысить 10-летнюю выживаемость пациентов с МРЩЖ [5]. Однако, несмотря на использование высокочувствительных лабораторных методов, частота ложноположительных результатов остается высокой, что определяет низкую положительную прогностическую значимость теста [6]. Ряд факторов и заболеваний может сопровождаться изменением уровня секреции кальцитонина. Так, к вторичной гиперкальцитонинемии могут приводить курение, С-клеточная гиперплазия, аутоиммунные заболевания щитовидной железы, почечная недостаточность, острый панкреатит, гипергастринемия, экстратиреоидные кальцитонин-секретирующие нейроэндокринные опухоли (мелкоклеточный рак легких, феохромоцитома, бронхиальный карциноид, нейроэндокринные опухоли поджелудочной железы и т.д.), меланома, рак молочной железы, колоректальный рак и ряд других состояний [7–9].

Информативность цитологического исследования в диагностике МРЩЖ значительно ниже по сравнению с дифференцированными формами карцином (60–70% против 90–95%) и в значительной мере зависит от опыта исследователя [10]. Основные трудности цитологической диагностики МРЩЖ обусловлены различными вариантами строения, способными «мимикрировать» под папиллярные, анапластические или фолликулярные формы карцином [11].

Таким образом, разнообразие гистологических типов медуллярных карцином, а также недостаточная специфичность определения сывороточного кальцитонина определяют актуальность поиска дополнительных, более информативных диагностических тестов. Определение кальцитонина в смывах из пункционной иглы (ТАБ-КТ) является перспективным методом, позволяющим повысить диагностическую точность изолированной ТАБ в диагностике как первичных форм МРЩЖ, так и рецидива/метастазов заболевания, снизив число ложноотрицательных и сомнительных результатов [12].

В отличие от высокодифференцированного рака щитовидной железы, при котором определение тиреоглобулина в смывах из пункционной иглы хорошо зарекомендовало себя в диагностике высокодифференцированных карцином, имеется мало данных об информативности определения и пороговом диагностическом уровне кальцитонина в смывах при МРЩЖ [3].

В обновленных клинических рекомендациях по МРЩЖ Американской тиреоидологической ассоциации (АТА) 2015 г. предложено определение ТАБ-КТ и иммуногистохимическое исследование образца при получении подозрительного цитологического заключения (уровень доказательности В) [3].

Ряд исследований показал высокую чувствительность и специфичность определения ТАБ-КТ в диагностике МРЩЖ, однако по-прежнему окончательно не определен пороговый диагностический уровень.

В данной работе впервые проведена оценка уровня и определен пороговый диагностический уровень ТАБ-КТ в российской популяции, а также проведена оценка точности определения ТАБ-КТ изолированно или вместе с ТАБ в диагностике как первичного МРЩЖ, так и рецидива/метастазов МРЩЖ в лимфатические узлы.

## ЦЕЛЬ

Первичной конечной точкой исследования была оценка диагностической точности определения кальцитонина в смывах из пункционной иглы при сравнении с цитологическим исследованием в диагностике МРЩЖ. Вторичной конечной точкой было определение оптимального диагностического уровня для использования в клинической практике.

## МЕТОДЫ

## Дизайн исследования

Ретроспективное сравнительное исследование по оценке диагностической значимости определения ТАБ-КТ в диагностике МРЩЖ у пациентов с узловыми изменениями/подозрительными лимфатическими узлами как с интактной щитовидной железой, так и с подозрением на рецидив/метастазы МРЩЖ. Исследованы данные пациентов, находившихся на обследовании и лечении в ФГБУ «НМИЦ эндокринологии» в период с 2015 по 2020 гг.

## Критерии соответствия

Критериями включения в исследование были узловые изменения в щитовидной железе, наличие подозрительных лимфатических узлов по данным УЗИ, индивидуальный или семейный анамнез спорадического МРЩЖ или МЭН-синдрома, высокий уровень сывороточного кальцитонина, а также наличие информации в истории болезни о выполненном смыве на кальцитонин из пункционной иглы.

Критериями исключения были вторичная гиперкальцитонинемия вследствие почечной недостаточности, нейроэндокринные опухоли легких или гастроинтестинального тракта, прием препаратов, интерферирующих с кальцитонином, гипергастринемия, декомпенсация углеводного обмена.

## Описание медицинского вмешательства

Изучены клинико-демографические и лабораторные данные пациентов с узловыми изменениями щитовидной железы и/или подозрительными лимфатическими узлами, повышенным уровнем базального или стимулированного кальцитонина как первично, так и у пациентов с индивидуальным анамнезом МРЩЖ. В исследование были включены только данные пациентов с гистологически подтвержденным МРЩЖ. У всех пациентов исходно оценивали базальный уровень кальцитонина, при получении сомнительных результатов (уровень кальцитонина менее 100 пг/мл) проводили стандартный стимулирующий тест с оценкой уровня кальцитонина после внутривенного введения глюконата кальция из расчета 2,5 мг/кг (0,27 мл/кг 10% раствора). У пациентов с интактной щитовидной железой также определяли уровень тиреоглобулина в смывах для исключения метастазов высокодифференцированных карцином. После получения образцов для цитологического исследования осуществляли забор смывов из пункционной иглы для определения уровня кальцитонина под УЗ-навигацией.

## Методы регистрации исходов

Инструментальное исследование

Ультразвуковое исследование щитовидной железы, лимфатических узлов центральных и латеральных/боковых треугольников шеи выполняли всем пациентам на аппарате экспертного класса VOLUSON E8 (General Electric, США). Оценку узловых изменений щитовидной железы проводили в соответствии со стандартизованной системой описания протоколов ультразвукового исследования EU-TIRADS (Thyroid Imaging Reporting and Data System). Выявленные изменения считали подозрительными при наличии следующих ультразвуковых характеристик: гипоэхогенная солидная структура, неровный, нечеткий или полициклический контур, точечные, гиперэхогенные включения (микрокальцинаты), преобладание переднезаднего размера узла над шириной («выше/чем/шире») [14]. При исследовании регионарных лимфатических узлов наиболее специфическими признаками, позволяющими заподозрить метастатическое поражение, считали наличие микрокальцинатов, кистозного компонента, периферической васкуляризации, сходство ткани лимфатического узла с тканью щитовидной железы, менее специфичными – увеличение размеров, закругленность контуров, отсутствие ворот [14].

Гормональное исследование

Забор образцов крови на кальцитонин производился в 8 ч утра натощак. Сывороточную концентрацию кальцитонина и уровень кальцитонина в смыве оценивали иммунохемилюминесцентным методом на автоматизированной системе Liaison XL (DiaSorin) с функциональной чувствительностью 3 пг/мл, пределы обнаружения и количественного определения базального кальцитонина 0,0–11,8 пг/мл у мужчин и 0,0–4,8 пг/мл у женщин.

Цитологическое исследование

ТАБ щитовидной железы выполняли под УЗ-навигацией с использованием тонкой иглы 22G. Сразу после аспирации и получения образца для цитологического исследования шприц промывали 0,5 мл физиологического раствора, полученный материал отправляли в лабораторию. Цитологическое исследование препаратов проводили патоморфологи ФГБУ «НМИЦ эндокринологии».

Аспирированный материал для цитологического исследования помещали на покровное стекло, высушивали и затем окрашивали по методу Романовского. Интерпретацию результатов цитологического исследования проводили в соответствии с международной системой классификации The Bethesda System for Reporting Thyroid Cytopathology 2017 г. Цитологическими критериями диагностики МРЩЖ были: многоклеточность, наличие полигональных, веретенообразных или эпителиоидных клеток, эксцентрично расположенное круглое ядро с «грубым» хроматином (неравномерность конденсации по типу «соли и перца»), дистрофические изменения в ядрах (анизонуклеоз), азурофильные цитоплазматические гранулы (при окрашивании по Май–Грюнвальду) и отложения амилоида.

Тиреоидэктомия и центральная лимфодиссекция с/без латеральной лимфодиссекции были выполнены пациентам, соответствующим следующим критериям: базальный уровень кальцитонина >100 пг/мл, уровень стимулированного кальцитонина >100 пг/мл, Bethesda IV, V, VI по данным цитологического исследования.

В соответствии с целями исследования на основании гистологического исследования и/или иммуногистохимического определения экспрессии кальцитонина все пациенты были классифицированы на две диагностические категории: доброкачественные и злокачественные изменения.

## Этическая экспертиза

В исследование были включены истории болезни пациентов ФГБУ «НМИЦ эндокринологии», которые добровольно в письменной форме выразили согласие на использование своей медицинской информации в научных целях.

## Статистический анализ

Статистический анализ проводили в пакете прикладных программ SPSSv23 Statistics (IBM Corporation, США). Анализ характеристических кривых (ROC-анализ) осуществляли в программе MedCalc, версия 19.6.4 (MedCalc Software, Бельгия).

В работе анализировали выборку объемом 67 наблюдений. Описательная статистика параметров, приводимых далее в таблицах, представлена как медианы, первый и третий квартили (Me [Q1; Q3]); n — объем анализируемой подгруппы, р — достигнутый уровень статистической значимости. Сравнение двух независимых выборок для количественных параметров осуществляли с использованием критерия Манна–Уитни (U-тест).

Определение диагностической точности проводили с помощью анализа характеристических кривых (ROC-анализ) и оценки площади под характеристической кривой (AUС ROC), диагностической чувствительности, диагностической специфичности для изолированной ТАБ и ТАБ-КТ. Оценку качества диагностической модели проводили в соответствии с экспертной шкалой для значений AUC. Оптимальные значения порога отсечения для ТАБ-КТ определяли на основании максимальной оценки диагностической чувствительности и диагностической специфичности теста, основанного на доле правильно классифицированных пациентов в соответствии с наибольшим значением индекса Юдена.

**Table:** Таблица 1. Уровень кальцитонина в сыворотке и смывах из пункционной иглыTable 1. Levels of calcitonin in serum and lavage from a puncture needle

Параметры		Ме [ Q1; Q3]	р
Базальный кальцитонин, пг/мл Муж. (0,0–11,8) Жен. (0,0–4,8)	Ме общая (n=67)	204,7 [ 1,0; 1310,0]	
МРЩЖ (n=52)	250,9 [ 7,1; 1310,00] М 174,7 [ 12,0; 791,0] Ж 291,2 [ 1,0; 1310,0]	р=0,02
Остальные гистологические диагнозы (n=15)	32,9 [ 1,0; 315,0]
ТАБ-КТ лимфатических узлов, пг/мл (n=30)	МРЩЖ	1516,7 [ 10,6; 2000,0]	p<0,001
Остальные гистологические диагнозы	9,4 [ 1,0; 26,8]
ТАБ-КТ щитовидной железы, пг/мл (n=37)	МРЩЖ	1896,1 [ 971,0; 2000,0]	p<0,001
Остальные гистологические диагнозы	1,0 [ 1,0; 3,87]

## РЕЗУЛЬТАТЫ

В исследование включены данные 67 пациентов (43 женщины, 24 мужчины), средний возраст составил 48 (17; 74) лет. Из общего числа пациентов у 28 было подозрение на МРЩЖ, из них 15 пациентов с интактной щитовидной железой и 13 пациентов с индивидуальным анамнезом МРЩЖ с подозрительными лимфатическими узлами и/или прогрессирующим нарастанием уровня кальцитонина.

По данным цитологического исследования характерные для МРЩЖ изменения выявлены в 28 случаях, высокодифференцированный рак щитовидной железы — в 10 случаях, в 8 случаях диагностированы доброкачественные изменения, в 4 случаях — фолликулярное новообразование, в 8 случаях — злокачественные изменения неуточненного гистогенеза, в 2 случаях получено неинформативное заключение, у 7 пациентов результаты цитологического исследования не были представлены. Следует отметить, что в одном случае неинформативного заключения материал был представлен белоксодержащей жидкостью, лимфоидными элементами разной степени зрелости, обнаружено несколько групп клеток, плохо просматриваемых, по которым было трудно судить о характере процесса. В другом случае — мазок малоклеточный с очень большой примесью крови, в связи с чем интерпретация клеточного материала была также затруднительна. В первом случае у пациента с подозрительными узловыми изменениями по данным УЗИ (TIRADS 5) и повышенным уровнем базального кальцитонина (56 пг/мл), но с неинформативной цитологией решение об оперативном вмешательстве было принято на основании данных высокого уровня кальцитонина в смыве (более 2000 пг/мл). Во втором случае неинформативное цитологическое заключение лимфатических узлов было получено у пациента с ранее выполненной тиреоидэктомией по поводу МРЩЖ, уровень кальцитонина в смыве превышал 2000 пг/мл. Диагноз МРЩЖ в обоих случаях был подтвержден гистологически. Поскольку исследование носило ретроспективный характер, в медицинских картах 7 пациентов с подозрительными изменениями по данным УЗИ (TIRADS 6) не были представлены заключения ранее выполненного цитологического исследования, однако с учетом наличия результатов ТАБ-КТ было принято решение о включении данных пациентов в исследование.

Окончательное гистологическое исследование подтвердило диагноз МРЩЖ у 52 пациентов. По данным цитологического исследования правильно классифицированы 27 (51,9%) пациентов с гистологически верифицированным МРЩЖ, у 5 (9,6%) пациентов по данным цитологии диагностирован высокодифференцированный рак щитовидной железы (ВДРЩЖ), у 8 (15,3%) — злокачественные изменения неясного гистогенеза, у 5 (9,6%) — доброкачественные изменения, у 2 (3,8%) — неинформативное заключение, у 5 (9,6%) пациентов результаты цитологии не были представлены.

У пациентов с гистологически верифицированным МРЩЖ медиана базального кальцитонина составила 250,9 [ 7,1; 1310,00] пг/мл, медиана ТАБ-КТ — 1777,4 [ 10,6; 2000,0] пг/мл, медиана ТАБ-КТ в смывах из лимфатических узлов — 1516,7 [ 10,6; 2000,0] пг/мл и 1896,1 [ 971,0; 2000,0] пг/мл из узлов щитовидной железы.

По данным ROC-анализа чувствительность и специфичность для ТАБ-КТ из лимфатических узлов составили 88,5 и 100% соответственно. AUC ROC составила 0,924 (95% ДИ 0,745–0,991) (рис. 1). Чувствительность изолированной ТАБ составила 72%, специфичность — 66%, AUC ROC для изолированной цитологии — 0,697 (95% ДИ 0,482–0,863) (рис. 1). Оптимальный диагностический уровень для значений ТАБ-КТ из лимфатических узлов составил 35,8 пг/мл (р<0,001).

**Figure:**
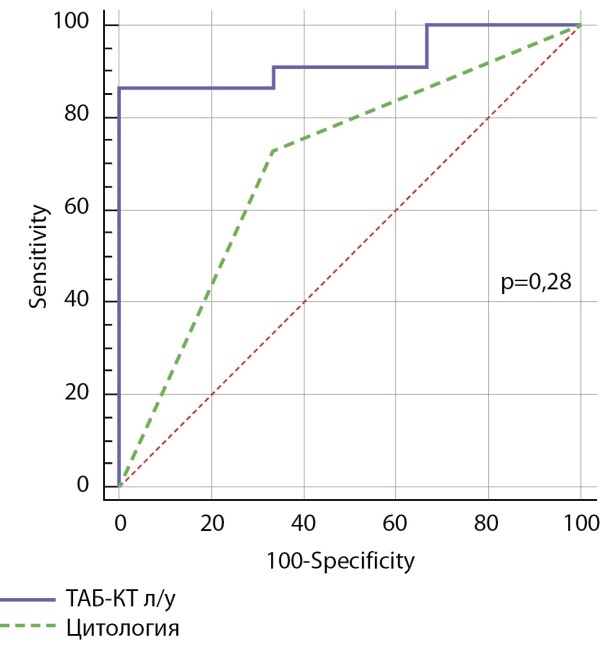
Рисунок 1. Сравнение ROC-кривых изолированной ТАБ и ТАБ-КТ из лимфатических узлов.Figure 1. Comparison of ROC-curves of isolated FAB and FNA-CT from lymph nodes.

Чувствительность и специфичность для ТАБ-КТ из узлов щитовидной железы составили 100% для обоих показателей соответственно, AUC ROC — 1,0 (95% ДИ 0,910–1,00) (рис. 2). Чувствительность изолированной ТАБ составила 67%, специфичность — 90%. AUC ROC для изолированной цитологии — 0,788 (95% ДИ 0,625–0,903) (рис. 2). Оптимальный диагностический уровень для значений ТАБ-КТ из узлов щитовидной железы составил 122 пг/мл (р<0,001).

**Figure:**
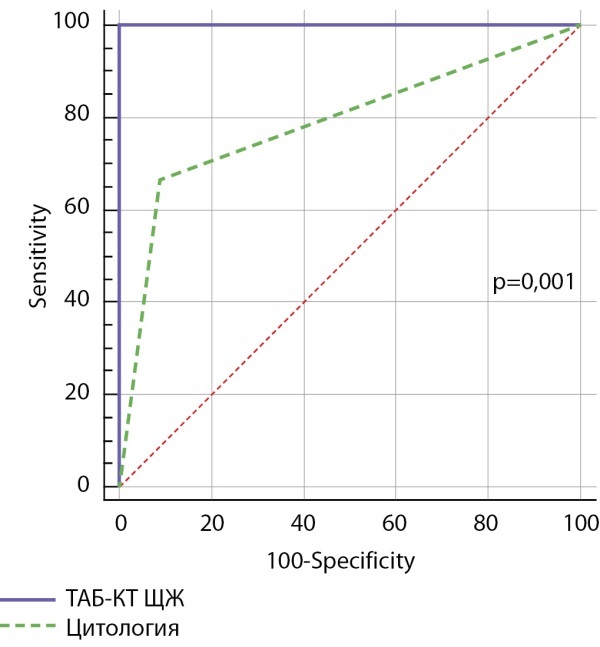
Рисунок 2. Сравнение ROC-кривых изолированной ТАБ и ТАБ-КТ из узлов щитовидной железы.Figure 2. Comparison of ROC-curves of isolated FAB and FAB-CT from thyroid nodules

## ОБСУЖДЕНИЕ

Своевременная и точная диагностика МРЩЖ важна для выявления как спорадических форм, так и карцином в рамках наследственных синдромов. Недостаточно высокая чувствительность цитологического исследования и определения сывороточного кальцитонина не всегда вовремя позволяет диагностировать и уточнить локализацию опухолевого процесса, что повышает риск нерадикального лечения. По аналогии с дифференцированными формами аденокарцином определение кальцитонина в смывах из пункционной иглы представляется перспективным методом диагностики МРЩЖ.

Первая работа по определению уровня кальцитонина в смывах из пункционной иглы в диагностике метастазов МРЩЖ была выполнена в 2007 г. В рамках исследования было показано, что 100% метастатически измененных узлов было выявлено с помощью ТАБ-КТ, в то время как только 62 и 80% имели положительный цитологический диагноз. В проспективном исследовании, сравнивающем ТАБ-КТ с определением базального кальцитонина, стимулирующим тестом с пентагастрином и цитологией, чувствительность составила 100% для ТАБ-КТ, 93,7% для определения базального кальцитонина, 87,5% для определения стимулированного кальцитонина и 12,5% для цитологии.

Результаты нашего исследования показали, что во всех случаях гистологически верифицированного МРЩЖ отмечаются повышенные уровни ТАБ-КТ как при первичных формах (чувствительность и специфичность 100%), так и при метастазах МРЩЖ в лимфатические узлы (чувствительность 88,5% и специфичность 100%). В то время как чувствительность и специфичность изолированной ТАБ щитовидной железы в диагностике первичного МРЩЖ составила 67 и 90%, а в диагностике метастазов в лимфатические узлы — 72 и 66% соответственно.

В нашем исследовании у 1 пациента с гистологически верифицированным папиллярным раком щитовидной железы определялся повышенный уровень базального кальцитонина (122 пг/мл). Выявленные изменения могут быть объяснены более чем в 2 раза высоким уровнем С-клеточной массы у пациентов с фолликулярными новообразованиями щитовидной железы по сравнению со здоровым контролем [12][13]. Данное предположение подтверждается также нормализацией уровня кальцитонина после тиреоидэктомии у пациентов с немедуллярным раком щитовидной железы.

Низкие значения ТАБ-КТ у пациентов с гистологически верифицированным МРЩЖ могут быть объяснены небольшими размерами образований (микрокарциномы), а также небольшим количеством высвобождаемого маркера в интерстициальную жидкость во время аспирации у пациентов с метастазами в лимфатические узлы и рецидивами МРЩЖ.

По-прежнему открытым остается вопрос оптимального уровня ТАБ-КТ в диагностике злокачественности. В исследованиях с гистологически подтвержденным МРЩЖ были получены различные пороговые диагностические значения для ТАБ-КТ (10,4, 36, 39,6, 67 пг/мл), определенные разными статистическими методами (ROC-анализ, использование 97,5 процентиля) [14–17]. В одном из исследований определяли уровень кальцитонина в смывах у пациентов без МРЩЖ или С-клеточной гиперплазии, исключенных гистологически или иммуногистохимически. Референсные значения ТАБ-КТ, определенные на основании 97,5 процентиля, в смывах с физиологическим раствором составили 8,5 пг/мл и 7,73 пг/мл в смывах с буфером для разведения образцов [17]. В нашем исследовании оптимальный диагностический уровень для определения злокачественности, рассчитанный методом ROC-анализа, составил 122 пг/мл из узлов щитовидной железы и 35,8 пг/мл из лимфатических узлов. Имеющаяся неоднородность в пороговых диагностических уровнях ТАБ-КТ, вероятно, возникает из-за различий в методологических аспектах проводимых исследований и исходной гетерогенности между пациентами, включаемыми в исследования.

## Ограничения исследования

Одним из основных ограничений нашего исследования является небольшой размер выборки. Проведение крупных исследований с большим количеством наблюдений позволит определить универсальный пороговый диагностический уровень. В отсутствие таких исследований целесообразна разработка локальных значений для диагностики злокачественности в подозрительных случаях.

Основной проблемой определения кальцитонина не в сыворотке или плазме крови является отсутствие подтвержденных экспериментальных данных, позволяющих валидировать результаты исследований, а также отсутствие формальной технической поддержки от производителей реактивов. Контаминация пункционного материала кровью с высоким содержанием кальцитонина может приводить к ложному повышению ТАБ-КТ, в таких случаях только уровень кальцитонина, значимо превышающий его сывороточную концентрацию, может рассматриваться в качестве диагностически значимого.

Поскольку определение уровня кальцитонина в смывах проводится с использованием иммуноаналитического метода, интерференция с гетерофильными антителами, обладающими высокой перекрестной реактивностью, может приводить как к повышению, так и снижению содержания аналита в образце [18]. Ложноотрицательные результаты также могут быть получены в случаях микрокарцином, а также при развитии hook-эффекта в случаях с очень высокой концентрацией кальцитонина, например, при диссеминированных формах МРЩЖ [19].

На аналитическом этапе на результаты исследования также могут влиять различия в антигенной структуре и иммунореактивности кальцитонина, а также матрикс-эффект жидкостей, использующихся для смыва. Однако, согласно данным литературы, использование физиологического раствора или буфера для разведения образцов существенным образом не влияет на результаты определения ТАБ-КТ [20].

## ЗАКЛЮЧЕНИЕ

Своевременная диагностика МРЩЖ на дооперационном этапе обусловлена необходимостью установления стадии заболевания и определения объема хирургического вмешательства. Наряду со ставшими традиционными диагностическими тестами (цитологическое исследование, определение базального и стимулированного кальцитонина) перспективным верифицирующим методом у пациентов с подозрением на МРЩЖ является определение уровня кальцитонина в смывах из пункционной иглы. Основные сложности исследования связаны с отсутствием точного порогового уровня для диагностики МРЩЖ, что в первую очередь обусловлено риском контаминации смывов кровью с высоким уровнем кальцитонина.

В данной работе представлен опыт ФГБУ «НМИЦ эндокринологии» по определению уровня кальцитонина в смывах из пункционной иглы. Главными преимуществами нашего исследования являются стратификация групп на основании гистологической верификации диагноза, а также сравнение с другими стандартными диагностическими процедурами (ТАБ и определение базального уровня кальцитонина). В качестве оптимального порогового диагностического уровня для определения злокачественности в узлах щитовидной железы предложены значения ТАБ-КТ>122 пг/мл, в лимфатических узлах — >35,8 пг/мл. В качестве превентивной стратегии ранней диагностики МРЩЖ представляется целесообразным определение уровня базального кальцитонина у пациентов, направляемых на ТАБ, с последующим определением уровня кальцитонина в смывах у пациентов с исходно повышенным уровнем базального кальцитонина в сыворотке. Для валидации диагностических значений ТАБ-КТ требуется проведение более крупных клинических исследований.

## ДОПОЛНИТЕЛЬНАЯ ИНФОРМАЦИЯ

Источники финансирования. Работа выполнена по инициативе авторов без привлечения финансирования.

Конфликт интересов. Авторы декларируют отсутствие явных и потенциальных конфликтов интересов, связанных с содержанием настоящей статьи

Участие авторов. Все авторы одобрили финальную версию статьи перед публикацией, выразили согласие нести ответственность за все аспекты работы, подразумевающую надлежащее изучение и решение вопросов, связанных с точностью или добросовестностью любой части работы.
